# Visual Attention and Aesthetic Evaluation of Two-Color TV-Cabinet Decorative Finishes in Older Adults

**DOI:** 10.3390/jemr19050107

**Published:** 2026-09-22

**Authors:** Dong Jin, Mengxian Wu, Ruishi Wang, Yiping Li, Xiaoxing Yan

**Affiliations:** College of Furnishings and Industrial Design, Nanjing Forestry University, Nanjing 210037, China; mengxianwu5945@163.com (M.W.); ruishiwang2025@163.com (R.W.); 15573782353@163.com (Y.L.); yanxiaoxing@njfu.edu.cn (X.Y.)

**Keywords:** age-friendly furniture, decorative finish, two-color TV cabinet, surface color, visual attention, subjective preference

## Abstract

For age-friendly furniture surface design, the color of a TV-cabinet finish affects not only visual comfort in the living room but also older users’ aesthetic preferences, perceived harmony, and emotional acceptance of furniture products. To clarify how older adults perceive hue, value, and the dominant–secondary area relationship in two-color decorative finishes for TV cabinets, this study used a digital model with a unified form and constructed surface color schemes varying in hue, value, and two-color dominance. The experiment contained two parts. Experiment 1 examined the differences in subjective evaluation and visual attention under colored hue × value conditions. Experiment 2 examined the changes in preference and attention under dominant/secondary color relationship × colored hue × value conditions. Thirty-five older participants completed an eye-tracking experiment and a seven-point semantic-differential questionnaire. The results showed that: (1) a three-item aesthetic scale comprising pleasure, simplicity, and order showed good internal consistency in both experiments; (2) hue had a significant effect on subjective pleasantness, while significant interactions indicated that preference also depended on specific combinations of hue, value, and color relationship—yellow showed relatively high descriptive pleasantness ratings under the tested conditions; and (3) the viewing duration varied with the experimental color factors, whereas no statistically significant associations were detected between subjective pleasantness and either the viewing duration or the fixation count under the tested conditions, suggesting that visual-attention measures and subjective preferences provide complementary rather than equivalent information. These findings provide preliminary empirical evidence from subjective evaluation and eye-movement data for further exploration of color configurations in age-friendly furniture decorative finishes and coating surfaces.

## 1. Introduction

Population aging has made age-friendly residential environments and furniture products important topics in furniture manufacturing, surface-decoration materials, and human-settlement research [1]. Furniture surface finishes, coating layers, and decorative films provide protection and durability, but they also influence users’ visual comfort and emotional evaluation through color, gloss, texture, and tactile qualities [2]. Existing studies on age-friendly furniture and decorative materials have indicated that older users’ visual, tactile, and emotional experiences are jointly affected by physiological changes and residential needs; therefore, furniture surface materials and color configurations should be evaluated with older users’ perceptual characteristics in mind rather than just from the perspectives of form or production convenience [3]. In the visual system, aging may be accompanied by changes in color discrimination, light adaptation, and visual-search efficiency, which can produce perceptual needs for furniture surface color, visual material attributes, and spatial color atmosphere that differ from those of younger users [4]. Huang et al. [5] examined older adults’ appraisal of visual–tactile associative imagery in smart products and showed that comfort, aesthetics, practical reliability, and tactile materials jointly affect acceptance among older users. Xue et al. [6] proposed that product design for older adults with perceptual limitations should reduce operational barriers in terms of vision, touch, button materials, and anthropometric dimensions. Lv [7] emphasized that age-friendly furniture should balance ease of use, psychological security, and emotional satisfaction according to older adults’ behavioral characteristics and psychological needs. Liu et al. [8], using kitchen cabinets as the research object, identified color and material as important entry points for improving older adults’ satisfaction with cabinet furniture, which provides a comparable basis for discussing TV-cabinet finish colors in this study.

TV cabinets are visually central and continuously exposed in domestic living rooms. Their surface-finish colors interact with the television background wall, storage function, living-room lighting, and older adults’ daily viewing behavior. Compared with monochromatic finishes, two-color finishes can enhance visual layering while maintaining an overall sense of order by changing local component colors, the dominant–secondary color relationship, and area proportion [9]. However, two-color schemes may also cause visual conflict, attention dispersion, or reduced preference when hue differences, value differences, or the colored area are excessive [10]. Therefore, studying older adults’ preferences and visual attention for the hue, value, and area relationships of two-color TV-cabinet decorative finishes can improve the theory of age-friendly furniture surface color design and provide actionable color strategies for furniture enterprises and developers of decorative coatings and finish materials.

### 1.1. Visual Perception of Decorative Surfaces in Age-Friendly Furniture

With the evolution of consumer demand, users no longer focus only on the structural performance and durability of furniture materials [11]; they also pay increasing attention to the visual, tactile, and emotional experiences created by furniture surfaces. Color is one of the most direct emotional cues in furniture decorative surfaces. Its effect is not determined by hue alone but by the combined action of value, saturation, gloss, texture, and background environment. A parametric color framework helps transform color from an experience-based design element into a controllable, comparable, and empirically verifiable research variable [12]. Huang et al. [13] analyzed the effects of color and texture combinations on visual perception in reconstituted decorative veneers and emphasized that furniture surface decoration is not governed by a single color factor. Zhou et al. [14] developed a wardrobe-furniture color-selection system linking color harmony and beauty evaluation with enterprise color selection, thereby providing a methodological reference for quantitative screening of furniture color schemes. Hu and Zhang [15] emphasized, from the perspective of furniture and interior decoration design, that furniture color should coordinate with the spatial atmosphere, use context, and overall decorative effect. Wei et al. [16] analyzed differences in consumer evaluation indicators for furniture appearance imagery and showed that material, color, style, form, and surface decoration jointly constitute furniture appearance imagery. Existing research has indicated that furniture surface color design has gradually shifted from single color attribute analysis to comprehensive perception research on the synergistic effects of multiple factors. Color not only affects users’ visual evaluation but is also closely related to material properties, spatial environments, and emotional experiences.

In color-combination research, Giraldo et al. [17] reviewed color theory and its relationship with older people’s moods, indicating that color influences older adults’ emotional state and perceptual response; this provides theoretical support for the present focus on color emotion among older users. Lee and Gong [18] discussed the relationship between color harmony and color emotions using two-color combinations applied to three-dimensional color configurations, demonstrating that two-color relationships are not simply the sum of two independent colors but generate an overall emotional evaluation. Dou and Xue [19], through image-based corpus analysis, further showed that color is not only a visual attribute but also participates in the construction of emotional meaning. In product color design and optimization, Guo et al. [20] used tricolor products as examples and treated color harmony and users’ emotional preferences as multi-objective optimization criteria, suggesting that product color matching should simultaneously consider area proportion, harmony, and emotional response. Hsiao and Peng [21] proposed a product color-matching method based on gradient colors and aesthetic measurement, and used eye-movement heat maps to screen design schemes, reflecting a data-assisted approach to product color design. Ding et al. [22] proposed a product color design method for brand-extension identification by generating product color schemes from a color-sample database, color genetic factors, and a genetic algorithm, showing that color design can support the extension of brand identity.

In the field of eye-tracking experimental research, Chen et al. [23] combined eye-tracking and PAD-based emotion measures to evaluate wardrobe-front colors and textures, showing that fixation behavior can objectively reflect material attractiveness and preference. Zhang et al. [24] used fixation duration, fixation count, and heat maps to assess visual-search efficiency, confirming eye tracking as an objective tool for analyzing visual attention. Overall, existing research has addressed older-user perception, furniture surface properties, two-color harmony, color layout, and eye-movement evaluation, but few studies have directly examined two-color furniture color schemes for older users while simultaneously comparing hue, value, colored-area proportion, subjective preference, and visual attention. Therefore, this study uses the TV cabinet as a living-room furniture item with high visual exposure, and combines eye tracking with a seven-point semantic-differential scale to analyze the effects of two-color TV-cabinet schemes on older users’ visual attention, subjective preferences, and overall harmony evaluation.

### 1.2. Present Study

Building on previous studies, this study integrates subjective evaluation, eye tracking, and statistical analysis to examine how hue, value, and dominant–secondary color–area relationships affect older adults’ subjective preferences, harmony evaluation, and visual attention toward two-color TV cabinets. Two experiments address: (1) the effects of hue and value on subjective preference; (2) the effects of dominant–secondary color–area relationships on visual attention and aesthetic evaluation; and (3) the consistency and complementarity between subjective ratings and eye-movement measures. The main contribution lies in integrating established methods within the specific context of age-friendly two-color furniture surfaces, providing empirical evidence for older adults’ color perceptions and age-friendly furniture surface color design.

## 2. Materials and Methods

### 2.1. Overall Study Design and Stimulus Construction

HSV is a perception-oriented color space comprising hue, saturation, and value. Compared with the RGB color model, HSV allows design researchers to control stimulus variables more conveniently in terms of hue, saturation, and value, and is more suitable for explaining users’ subjective perceptions of color appearance. In this study, hues and values were controlled within the HSV color framework, and the HSV parameters were converted to RGB values for screen presentation using a standard HSV-to-RGB conversion procedure. During conversion, H was expressed on a 0–360° scale, while S and V were normalized to 0–1, and the resulting RGB components were subsequently mapped to the 8-bit RGB range (0–255). The same conversion procedure was applied to all experimental stimuli to ensure consistency in color generation and screen presentation [25,26].

The IKEA BESTÅ TV cabinet with doors (Nanjing, China) was selected as the experimental prototype. According to sales data from the IKEA Nanjing store over the past year, the series achieved cumulative sales of more than 23,000 units, indicating broad market acceptance and user coverage. Its clearly defined finish zones on the cabinet body, door panels, and legs facilitated control of two-color variables while maintaining a consistent product form. Accordingly, the form, viewing angle, background, lighting, and size were held constant, with only the two-color surface-finish configuration being varied. Hues were selected based on furniture-market research and the HSV hue circle. Thirty-six candidate hues at 10° intervals were ranked by seven design students for recognizability, representativeness, and suitability for furniture surfaces, followed by a review by three experts. Five hues were ultimately selected: red (0°), orange (20°), yellow (50°), green (150°), and blue (200°). Value was set at V = 22%, 55%, and 88%, representing low, medium, and high levels, respectively [27], with saturation fixed at 100%. White served as the achromatic base for the dominant–secondary color relationship. The frontal colored-to-noncolored area ratios were 6.15%:93.85% for the small-area secondary-color condition and 55.98%:44.02% for the large-area dominant-color condition; visible proportions could vary slightly because of the oblique viewing angle. The digital model controlled potential interference from form, material, and lighting; therefore, the results represent a visual simulation of decorative-finish colors rather than physical testing of actual coating samples.

Two connected within-subject eye-tracking experiments were conducted. Experiment 1 tested five hues × three value levels under large-area colored-finish conditions, yielding 15 conditions. Experiment 2 added the dominant/secondary color relationship, combining two color-role structures with the same five hues and three value levels, yielding 30 conditions. The overall factorial structure and stimulus configuration of the two eye-tracking experiments are summarized in Table 1. The two experiments used the same TV-cabinet form, viewing equipment, participant sample, subjective scales, and eye-movement indicators to ensure comparability.

As shown in Figure 1, each stimulus image contained four TV cabinets corresponding to four areas of interest (AOIs), and the size and shape of each AOI were basically consistent. To enable repeated measurement of the same color factors under different control conditions, the presentation order of the samples followed a Latin square design throughout the experiment. In Experiment 1, the 15 samples appeared repeatedly four times, and in Experiment 2, the 30 samples appeared repeatedly twice, forming 15 stimulus pages in each experiment. Eye-movement analysis used fixation duration, fixation count, and within-page normalized proportions within each AOI as the core indicators.

### 2.2. Common Experimental Conditions

#### 2.2.1. Participants and Ethics

Thirty-five healthy older adults were recruited for this experiment (15 males, 20 females). The participants were aged 60–76 years, with a mean age of 65.63 years (SD = 4.60 years). The sample size was determined by a priori power analysis using G*Power 3.1. Assuming a medium effect size (f = 0.25), a significance level of α = 0.05, and statistical power 1 − β = 0.80, the minimum required sample size for repeated-measures analysis of variance (RM-ANOVA) was calculated to be 28. Accordingly, the 35 participants in the present study met the statistical requirements. All participants had normal or corrected-to-normal visual acuity and normal color vision, which was screened using Ishihara Pseudoisochromatic Plates published by People’s Medical Publishing House. The study protocol was approved by the Ethics Committee of Nanjing Forestry University. All participants were fully informed of the experimental procedures, provided written informed consent prior to the experiment, and received corresponding remuneration upon completion of the experiment.

#### 2.2.2. Apparatus

The eye-tracking experiment was conducted using a Tobii Pro Fusion screen-based eye tracker (Tobii Technology, Stockholm, Sweden) mounted below a Lenovo monitor, with the sampling rate set to 30 Hz. Participant data were recorded and processed using the ErgoLAB 3.0 eye-tracking analysis platform. The host computer operated on Windows 11 and was equipped with a 1920 × 1080-pixel LCD monitor (Lenovo, Nanjing, China). Monitor brightness and display parameters were uniformly calibrated before the experiment and kept constant throughout the testing procedure. In a controlled laboratory environment with constant illumination, participants were seated approximately 65 cm from the display and completed a standard nine-point calibration procedure after adopting a comfortable sitting posture. Calibration quality was indicated by a progress bar that gradually changed from red to green; formal testing commenced once the indicator turned green, indicating satisfactory calibration quality. Upon completion of the eye-tracking task, participants completed a semantic-differential questionnaire to rate their perceptions and preferences for each sample. During data processing, fixation metrics, including total fixation duration and fixation count, were calculated using the Velocity-Threshold Identification (I-VT) algorithm implemented in ErgoLAB 3.0, which distinguishes fixations from saccades based on gaze velocity. The I-VT’s velocity threshold was set to 30°/s, and the minimum fixation duration was set to 60 ms. The same parameters were applied consistently across all participants and experimental conditions. Only eye-movement data assigned high-confidence validity labels by the eye-tracking system were included in the final analysis.

#### 2.2.3. Measurement Metrics

Subjective evaluation used a seven-point semantic-differential scale ranging from −3 to +3. The five adjective pairs were cool–warm, heavy–light, unpleasant–pleasant, complex–simple, and disorderly–orderly. The unpleasant–pleasant dimension was used as the core indicator of subjective preference. Pleasure, simplicity, and order jointly formed a three-item aesthetic composite indicator reflecting overall harmony evaluation.

Eye-movement indicators included total fixation duration, fixation count, within-page proportion of fixation duration, and within-page proportion of fixation count. Because stimulus-page viewing was self-paced, total dwell time could vary across participants and pages. To reduce the influence of this variability, fixation duration and fixation count were analyzed together with their within-page normalized proportions for each AOI. The normalized measures therefore reflected the relative allocation of attention within each stimulus page rather than absolute viewing time alone. Heat maps were divided according to the TV-cabinet geometry into four regions: top panel, open shelf/upper part, door panel, and leg/bottom region. All eye-movement indicators were interpreted as evidence of visual attention and processing rather than direct equivalents of subjective preference.

#### 2.2.4. General Experimental Procedure

The experimental procedure consisted of sign-in, eye-tracker calibration, formal image-viewing task, and subjective questionnaire evaluation. As illustrated in Figure 2, participants were instructed to observe freely and click the mouse to select their preferred color scheme on each stimulus page for page transition. A self-paced blank page was presented between consecutive stimulus images, and participants proceeded to the next stimulus by clicking the mouse when ready. Upon completion of the eye-tracking task, participants rated the corresponding samples using the seven-point semantic-differential scale.

### 2.3. Experiment 1: Colored Hue × Value

#### 2.3.1. Experimental Purpose and Factor Design

Experiment 1 aimed to identify the basic effects of colored hue and value on older users’ visual attention and aesthetic evaluation. A 5 (hue: red, orange, yellow, green, blue) × 3 (value: 22%, 55%, 88%) within-subject design was used. In Experiment 1, the colored finish was located in a large visible area of the TV cabinet, with white serving as the secondary color, resulting in 15 color-matching conditions. Dependent variables included five semantic evaluation indicators, total fixation duration, fixation count, and within-page normalized attention proportions.

#### 2.3.2. Stimulus Materials and Presentation

All stimuli in Experiment 1 were produced using the same digital model of the IKEA BESTÅ TV cabinet with doors. The form, viewpoint, background, lighting, and size were kept constant, and only the hue and value of the colored finish were changed. Each stimulus page contained four TV-cabinet samples corresponding to four areas of interest (AOIs), with the size and shape of the AOIs kept approximately consistent. To obtain repeated measurements for each color condition, each of the 15 experimental conditions was presented four times in Experiment 1, with the presentation order arranged according to a Latin square design.

#### 2.3.3. Experimental Procedure and Data Collection

Experiment 1 followed the common procedure. After eye-tracker calibration, participants sequentially viewed 15 stimulus pages representing the 15 hue × value conditions. The system recorded fixation duration and fixation count within each AOI. After the viewing task, participants evaluated the Experiment 1 samples on cool–warm, heavy–light, unpleasant–pleasant, complex–simple, and disorderly–orderly scales.

#### 2.3.4. Statistical Analysis

Statistical analysis of the experimental data was executed using SPSS 27.0 software. In Experiment 1, descriptive statistics were first calculated for subjective evaluations and eye-movement measures. Given the within-subject repeated-measures design, subjective pleasantness was analyzed using a random-intercept linear mixed-effects model (LMM) to account for the dependency of repeated observations within participants. Participant was specified as a random intercept, while hue, value, and their interaction were included as fixed effects. Omnibus fixed effects were evaluated using Wald χ^2^ tests, and significant effects were followed by pairwise comparisons of estimated marginal means with Holm adjustment for multiple comparisons. Eye-movement outcomes were analyzed using two-way repeated-measures ANOVA with hue and value as within-subject factors. Greenhouse–Geisser correction was applied when the assumption of sphericity was violated, and partial η^2^ was reported as the effect size. Associations between subjective pleasantness and eye-movement measures were examined using within-subject correlations to account for the repeated-measures structure within participants.

### 2.4. Experiment 2: Dominant/Secondary Color Relationship × Hue × Value

#### 2.4.1. Experimental Purpose and Factor Design

Experiment 2 aimed to test whether the dominant–secondary relationship between colored and white areas in the furniture structure moderated the effects of hue and value. A 2 (dominant/secondary color relationship) × 5 (hue: red, orange, yellow, green, blue) × 3 (value: 22%, 55%, 88%) within-subject design was adopted. The two color relationships were the colored finish as the dominant color with white as the secondary color and white as the dominant color with the colored finish as the secondary color, yielding 30 experimental conditions.

#### 2.4.2. Stimulus Materials and Presentation

All stimuli in Experiment 2 were generated using the same digital model of the IKEA BESTÅ TV cabinet with doors and the same color parameters as in Experiment 1. The furniture form, viewing angle, background, lighting, and dimensions were held constant, while the role of the colored finish as the dominant or secondary color was additionally manipulated. When the colored finish served as the dominant color, it occupied a relatively large visible area; when white served as the dominant color, the colored finish was used as a small-area accent. Each stimulus page contained four TV-cabinet samples corresponding to four areas of interest (AOIs), with the size and shape of the AOIs kept approximately consistent. To obtain repeated measurements for each color condition, each of the 30 experimental conditions was presented twice in Experiment 2, with the presentation order arranged according to a Latin square design.

#### 2.4.3. Experimental Procedure and Data Collection

Experiment 2 was conducted under the same apparatus, environment, and participant conditions as Experiment 1. After eye-tracker calibration, participants sequentially viewed 15 stimulus pages representing 30 dominant–secondary color relationship × hue × value conditions. The eye-tracking system synchronously recorded fixation duration, fixation count, and their within-page proportions within each AOI. After viewing, participants completed subjective evaluation using the same seven-point semantic-differential scale.

#### 2.4.4. Statistical Analysis

Statistical analysis of the experimental data was executed using SPSS 27.0 software. Descriptive statistics were first calculated for subjective evaluations and eye-movement measures. Consistent with Experiment 1, LMM was used as the primary inferential framework. Subjective pleasantness, dominant/secondary color relationship, hue, value, and their interactions were specified as fixed effects, with participant included as a random intercept. Eye-movement outcomes were analyzed using three-way repeated-measures ANOVA, with dominant/secondary color relationship, hue, and value as within-subject factors. Greenhouse–Geisser correction was applied when the assumption of sphericity was violated, and partial η^2^ was reported as the effect size. Omnibus fixed effects were evaluated using Wald χ^2^ tests, and significant effects were followed by pairwise comparisons of EMMs with the Holm adjustment for multiple comparisons. Associations between subjective pleasantness and eye-movement measures were examined using within-subject correlations.

### 2.5. Cross-Experiment Common Analysis

Across both experiments, the three-item aesthetic scale (pleasure, simplicity, and order) was evaluated using Cronbach’s alpha, McDonald’s omega, KMO, Bartlett’s test, single-factor explained variance, and AVE. Cross-experiment analyses further examined the consistency of subjective evaluations and eye-movement patterns between Experiments 1 and 2. For the primary LMM analyses, omnibus Wald χ^2^ tests were unadjusted, whereas post hoc pairwise comparisons were Holm-adjusted.

## 3. Results

### 3.1. Reliability and Structural Validity of the Common Scale

As shown in Table 2, the three-item aesthetic scale demonstrated good internal consistency, with Cronbach’s α/McDonald’s ω values of 0.884/0.929 in Experiment 1 and 0.878/0.926 in Experiment 2. The corresponding KMO values were 0.616 and 0.591, and Bartlett’s tests were significant in both experiments (*p* < 0.001). A single factor explained more than 80% of the variance, with AVE values exceeding 0.80. Given the relatively low KMO value in Experiment 2, the use of only three items, and the limited sample size (*n* = 35), the factor-analytic findings should be regarded as exploratory. Nevertheless, the results provide preliminary support for using pleasantness, simplicity, and orderliness as a composite aesthetic–evaluation index in the present study.

### 3.2. Experiment 1: Hue × Value

#### 3.2.1. Effects of Hue and Value on Visual Attention

Experiment 1 showed that hue had a significant effect on total fixation duration, with F (4, 136) = 7.026, *p* < 0.001 after Greenhouse–Geisser correction, and partial eta squared = 0.171. The red condition had the longest viewing duration (M = 0.606 s), followed by orange (M = 0.472 s) and yellow (M = 0.450 s), whereas the green condition had the lowest value (M = 0.406 s). The main effect of value was also significant, F (2, 68) = 10.036, corrected *p* < 0.001, partial eta squared = 0.228. The hue × value interaction was significant, F (8, 272) = 3.308, *p* = 0.001, partial eta squared = 0.089, indicating that the effect of value on visual attention varied by hue.

#### 3.2.2. Effects of Hue and Value on Subjective Evaluation

Descriptive statistics showed that yellow received the highest mean pleasantness rating (M = 0.87), followed by blue (M = 0.59), red (M = 0.58), green (M = 0.44), and orange (M = 0.31) (Figure 3). The 95% confidence intervals showed substantial overlap across hue conditions; therefore, the ordering of the marginal means should be interpreted as a descriptive pattern rather than as evidence of significant differences between specific hues. The linear mixed-effects model further revealed a significant omnibus effect of hue, Wald χ^2^ (4) = 12.43, *p* = 0.014, whereas the main effect of value was not significant, Wald χ^2^ (2) = 4.01, *p* = 0.135. A significant hue × value interaction was also observed, Wald χ^2^ (8) = 29.18, *p* < 0.001, indicating that the effect of value on pleasantness varied across hues. Holm-adjusted pairwise comparisons showed that yellow was rated significantly higher than orange (adjusted *p* = 0.009), whereas the remaining hue comparisons were not significant. Overall, subjective pleasantness was better characterized by specific hue–value combinations than by the descriptive ranking of hue means alone.

As shown in Figure 4, pleasantness ratings in Experiment 1 varied across hue × value combinations, consistent with the significant hue × value interaction identified by the LMM. Yellow showed relatively high and stable ratings across the three value levels, while red also remained relatively stable. In contrast, orange and green showed greater variation across value levels, whereas blue displayed an increasing trend with value. These patterns indicate that the association between value and subjective pleasantness differed across hues instead of reflecting a uniform value effect. Therefore, Figure 4 should be interpreted primarily as an interaction pattern between hues and values.

### 3.3. Experiment 2: Color Relationship × Hue × Value

#### 3.3.1. Main Effects on Visual Attention

Experiment 2 reconstructed the two-color relationship from the original area-proportion description into a dominant/secondary color relationship. The results indicated that colored hue, value, and dominant–secondary relationship all affected total fixation duration. The main effect of hue was significant, F (4, 136) = 21.723, corrected *p* < 0.001, partial eta squared = 0.390; red (M = 0.661 s) and blue (M = 0.598 s) produced longer viewing durations, while green was lower (M = 0.331 s). The main effect of value was significant, F (2, 68) = 4.261, corrected *p* = 0.019, partial eta squared = 0.111. The main effect of dominant–secondary relationship was also significant, F (1, 34) = 45.191, *p* < 0.001, indicating that using color as the dominant tone significantly increased surface visual exposure. Given the significant higher-order interactions, these main effects should be interpreted in conjunction with the specific combinations of hue, value, and color–area relationship.

As shown in Figure 5, the marginal means averaged across hue and color–area conditions showed that both the fixation-duration share and fixation-count share increased from the low-value condition (22%) to the medium-value condition (55%). At the high-value level (88%), fixation-duration share remained relatively high, whereas the fixation-count share decreased slightly compared with the medium-value condition. These aggregated patterns descriptively indicate differences in visual-attention allocation across value levels. However, given the significant higher-order interactions, the marginal trends should be interpreted descriptively rather than as evidence of a uniform value effect across hue and color–area conditions.

#### 3.3.2. Interaction Effects on Subjective Preference

The LMM showed a significant overall effect of hue (Wald χ^2^ (4) = 54.16, *p* < 0.001), whereas the main effects of value and color–area proportion were not significant (*p* = 0.891 and *p* = 0.940, respectively). Significant interactions were observed for hue × value (Wald χ^2^ (8) = 20.35, *p* = 0.009), hue × color–area proportion (Wald χ^2^ (4) = 24.39, *p* < 0.001), value × color–area proportion (Wald χ^2^ (2) = 6.54, *p* = 0.038), and the three-way interaction (Wald χ^2^ (8) = 23.06, *p* = 0.003). These results indicate that subjective preference for two-color TV-cabinet finishes depended on specific combinations of hue, value, and color–area proportion rather than on their independent effects alone.

As shown in Figure 6, under the small-area accent-color condition, pleasantness varied across hue × value combinations. Yellow showed relatively high ratings and reached the highest mean pleasantness score at the high-value level (M = 1.371), whereas blue remained relatively low across value levels. Red showed comparatively stable positive ratings, while orange and green exhibited greater variation across value levels. This pattern is consistent with the significant interaction effects identified by the LMM, and it suggests that the preference associated with value depended on the specific hue and color–area condition.

### 3.4. Cross-Experiment Analysis

#### 3.4.1. Consistency Between Subjective Preference and Eye-Movement Indicators

The correlation analysis between subjective pleasure and eye-movement indicators is shown in Table 3. The participant served as the repeated-measures unit (*n* = 35), and within-subject r accounted for repeated observations within participants. Condition-mean rho was computed across the condition means and is reported only as a complementary descriptive analysis; the two analysis levels were not treated as equivalent or independent. The results showed no stable or significant correlations between subjective pleasure and viewing duration, fixation count, or their within-page proportions. In Experiment 1, the within-subject correlation between viewing duration and pleasure was r = 0.057 (*p* = 0.193), and the correlation between fixation count and pleasure was r = 0.032 (*p* = 0.470). In Experiment 2, the correlation between viewing duration and pleasure was r = −0.010 (*p* = 0.754), and that between fixation count and pleasure was r = −0.010 (*p* = 0.749). Combined with Figure 7, each point represents the mean of one experimental color condition across participants, with the numeric label identifying the corresponding stimulus condition. These condition-mean associations are descriptive complements to the within-subject correlation analysis and should not be treated as independent participant-level observations. These results indicate that the present data did not detect a stable linear association between subjective pleasantness and the two eye-movement indicators under the tested conditions, rather than demonstrating the absence of any relationship.

#### 3.4.2. Heat Maps and Regional Distribution of Visual Attention

The normalized hotspot map shows that the visual attention of elderly people in the evaluation process of TV cabinets is not evenly distributed, but is influenced by the combination of furniture structure areas and color decoration spatial layout. Table 4 presents the participant-level mean proportions of fixation duration across different TV-cabinet regions (*n* = 35). Regional differences were evaluated using a within-subject analysis, with the overall regional effect reflecting differences across all structural regions rather than pairwise differences between specific regions. In Experiment 1, the distribution of viewing duration among regions was significant (F = 6.714, *p* = 0.001, ηp^2^ = 0.165), with the top-panel region receiving the highest proportion of attention (34.5%), followed by the leg region (23.9%), open shelf (21.2%), and door panel (20.4%). In Experiment 2, regional attention distribution remained significant (F = 6.831, *p* = 0.001, ηp^2^ = 0.167). The door-panel region received the highest viewing-duration proportion (32.6%), followed by the top panel (29.8%), open shelf (19.5%), and leg region (18.1%). Compared with Experiment 1, the increased attention toward the door-panel region in Experiment 2 suggests that when the colored finish occupied a larger visual area and became the dominant color region, it enhanced the visual salience of the corresponding surface. These results indicate that older adults’ visual attention was affected not only by the inherent structural hierarchy of furniture but also by the distribution and dominance of surface color information. It should be noted that the four cabinet regions differ in size, geometry, and position. The percentages in Table 4 represent each region’s share of total fixation duration and were not normalized by AOI. Therefore, these values describe the distribution of visual attention across regions rather than serving as direct measures of perceptual salience.

#### 3.4.3. Subjective Evaluation and Within-Subject Associations

Given the within-subject repeated-measures design, random-intercept linear mixed-effects models (LMMs) were used to examine the effects of color factors on subjective pleasantness, with participant specified as a random intercept. As shown in Table 5, in Experiment 1, the main effect of hue was significant (Wald χ^2^ (4) = 12.43, *p* = 0.014), and the hue × value interaction was also significant (Wald χ^2^ (8) = 29.18, *p* < 0.001), whereas the main effect of value was not significant (Wald χ^2^ (2) = 4.01, *p* = 0.135). In Experiment 2, the main effect of hue was significant (Wald χ^2^ (4) = 54.16, *p* < 0.001), whereas the main effects of value and color–area proportion were not significant (both *p* > 0.05); all two-way interactions and the three-way interaction were significant (all *p* < 0.05). These results indicate that subjective pleasantness was influenced by specific combinations of color factors rather than solely by their independent effects. The *p* values reported in Table 5 are unadjusted omnibus Wald χ^2^ test results for the fixed effects; the Holm adjustment was applied only to post hoc pairwise comparisons following significant omnibus effects.

As shown in Figure 8, within-subject correlation analyses were conducted to examine the relationships between the five adjective-pair evaluations and eye-movement indicators. Pleasantness was positively associated with warmth, lightness, simplicity, and orderliness in both experiments, with the strongest association observed for lightness (Experiment 1: r = 0.44; Experiment 2: r = 0.49). Viewing duration and fixation count were strongly correlated with each other (Experiment 1: r = 0.89; Experiment 2: r = 0.96) but generally showed limited associations with semantic evaluations. Except for a weak positive association between simplicity and viewing duration in Experiment 1 (r = 0.105, *p* = 0.016), no other stable associations with viewing duration or fixation count were detected. These findings suggest that semantic evaluations were more closely associated with integrated color perception and visual-order judgments than with individual fixation behaviors, supporting a distinction between visual attention and subjective aesthetic evaluation.

## 4. Discussion

### 4.1. Hue-Dependent Preferences for Two-Color TV Cabinets

Under the controlled digital-display conditions, hue showed a relatively strong association with subjective preference, although its effect depended on its interactions with value and color–area relationship. Yellow showed relatively high aesthetic acceptance, particularly in some small-area secondary-color conditions. These findings suggest that high-value yellow may be a potential color option worth further exploration in age-friendly two-color furniture rather than a direct design prescription.

### 4.2. Combined Effects of Value and Color–Area

The analyses of variance in both experiments showed that value affected viewing duration, whereas subjective pleasantness did not exhibit a consistent trend across value levels. In Experiment 2, large-area color conditions resulted in longer viewing durations, while the LMM showed that the main effect of the dominant–secondary color relationship on pleasantness was not significant, but its interactions with hue and value were significant. These findings suggest that color–area relationships may affect visual attention and aesthetic preference differently, with preference depending more on specific combinations of hue, value, and color–area. Therefore, these factors should be considered jointly in furniture-finish design rather than as independent determinants of preference. This is consistent with Li et al. [10], who reported that differences in hue, value, and chroma jointly influence color harmony and preference in two-color combinations. The study by Ding and Dong [28], which emphasized the importance of color layout in emotional product color design, also supports considering the dominant–secondary color relationship as an important factor influencing visual attention and aesthetic order.

### 4.3. Divergence Between Visual Attention and Subjective Aesthetic Preference

In this study, viewing duration and fixation count were not significantly correlated with subjective pleasure, indicating that the present data did not detect a stable linear association between eye-movement measures and subjective aesthetic evaluation under the tested conditions. Importantly, the absence of statistically significant correlations should not be interpreted as evidence that no relationship exists. This finding is consistent with Ji et al. [29], who combined eye-movement data with emotional evaluation and aesthetic ratings. One possible explanation lies in the distinction between bottom-up and top-down attention. Visually salient colors, such as red, may attract longer viewing because of bottom-up attentional capture, comparison among alternatives, or greater processing demands, rather than stronger preference. However, because attentional capture and cognitive load were not directly measured, these mechanisms should be regarded as possible explanations rather than confirmed causes. Thus, eye-movement measures may primarily reflect visual attention and processing, whereas subjective ratings reflect aesthetic evaluation, and the two may provide complementary rather than equivalent information. This interpretation is also supported by previous product-evaluation studies using eye tracking [30,31], which suggest that eye-movement measures can provide objective evidence of visual-attention processes but should be interpreted together with subjective evaluations.

After matching the five adjective pairs with preference and eye-movement indicators, this study further found that older adults’ preference judgments did not simply come from fixation duration or fixation count but were closely associated with semantic evaluation dimensions, such as lightness, simplicity, order, and warmth. This suggests that two-color TV-cabinet color design should not only pursue color salience but also reduce visual heaviness and complexity, enabling the color relationship to form a clear, stable, and emotionally warm overall impression. For older users especially, visual comfort and order may promote positive preference more effectively than strong visual stimulation.

### 4.4. Implications for Age-Friendly TV-Cabinet Color Design

Within the controlled digital-display conditions examined in this study, yellow showed relatively favorable subjective evaluations, and high-value yellow used as a small-area accent may be considered a potential option for further exploration in age-friendly two-color furniture. The findings also suggest that greater visual attention does not necessarily correspond to greater subjective preference, highlighting the complementary roles of subjective evaluation and eye-movement measures in understanding color responses. These findings should be regarded as preliminary design evidence rather than general design rules. Their applicability to physical furniture should be further evaluated under different lighting, gloss, texture, material, and surrounding color conditions.

### 4.5. Limitations and Future Research

This study has several limitations. First, screen-presented digital models were used, whereas surface properties such as gloss and texture, as well as ambient lighting and tactile experience, may alter color perception in physical furniture. Therefore, the findings should be regarded as preliminary empirical evidence for decorative-finish color configuration rather than as direct design recommendations for physical furniture. Second, although saturation, display settings, and ambient lighting were controlled, the CIELAB coordinates and absolute luminance of the stimuli were not directly measured; thus, residual differences in perceived lightness or chroma across hues cannot be completely excluded. Third, because four TV-cabinet alternatives were presented simultaneously on each stimulus page, fixation behavior may partly reflect comparative visual search and competition among alternatives rather than isolated responses to a single color condition. Self-paced viewing may also introduce individual differences in page-level viewing duration. Although the present study additionally analyzed within-page normalized proportions of fixation duration and fixation count alongside the raw eye-movement measures to partially reduce the influence of this variability, residual effects associated with differences in viewing time cannot be completely excluded. In addition, potential differences associated with age subgroups, visual abilities, and residential experience were not further examined. Future studies should incorporate colorimetric measurements, physical furniture finishes, and varied environmental conditions to further evaluate the reproducibility and practical applicability of these findings.

## 5. Conclusions

This study combined eye tracking and subjective questionnaires to examine older adults’ visual attention and aesthetic preferences for hue, value, and dominant–secondary area relationships in two-color TV-cabinet decorative surfaces. The main conclusions are as follows.

(1) The three-item aesthetic scale comprising pleasure, simplicity, and order showed good internal consistency and basically acceptable exploratory structural validity in both experiments, and can be used to reflect older adults’ overall aesthetic and harmony evaluation of two-color TV-cabinet decorative surfaces.
(2) Within the controlled digital-display conditions and color range examined in this study, yellow showed relatively high preference among older adults, with high-value yellow exhibiting favorable evaluations in some small-area secondary-color conditions. These results provide preliminary evidence for the potential use of yellow in age-friendly two-color furniture surfaces.
(3) Viewing duration and fixation count were not stably and significantly correlated with subjective pleasure, indicating that visual attention cannot directly substitute for aesthetic preference and should instead be used as supplementary evidence of visual attraction and processing.
(4) Heat maps and regional analyses showed significant differences in older adults’ visual attention across TV-cabinet structural regions, and the positions of colored finishes jointly affected visual distribution with the furniture structure.
(5) In design applications, high-value yellow used as a small-area accent may be considered a potential option for further exploration in two-color finishes for age-friendly TV cabinets. Large-area color applications should be evaluated in combination with hue and value, particularly because greater visual attention did not necessarily correspond to greater subjective preference.

This study provides preliminary empirical insights into older adults’ visual preferences for furniture decorative surfaces and offers a reference for further research on color configuration in age-friendly furniture. The findings suggest how two-color finishes may influence visual attention and aesthetic evaluation under controlled digital-display conditions, providing an empirical basis for further investigation of age-friendly furniture color design.

## Figures and Tables

**Figure 1:**
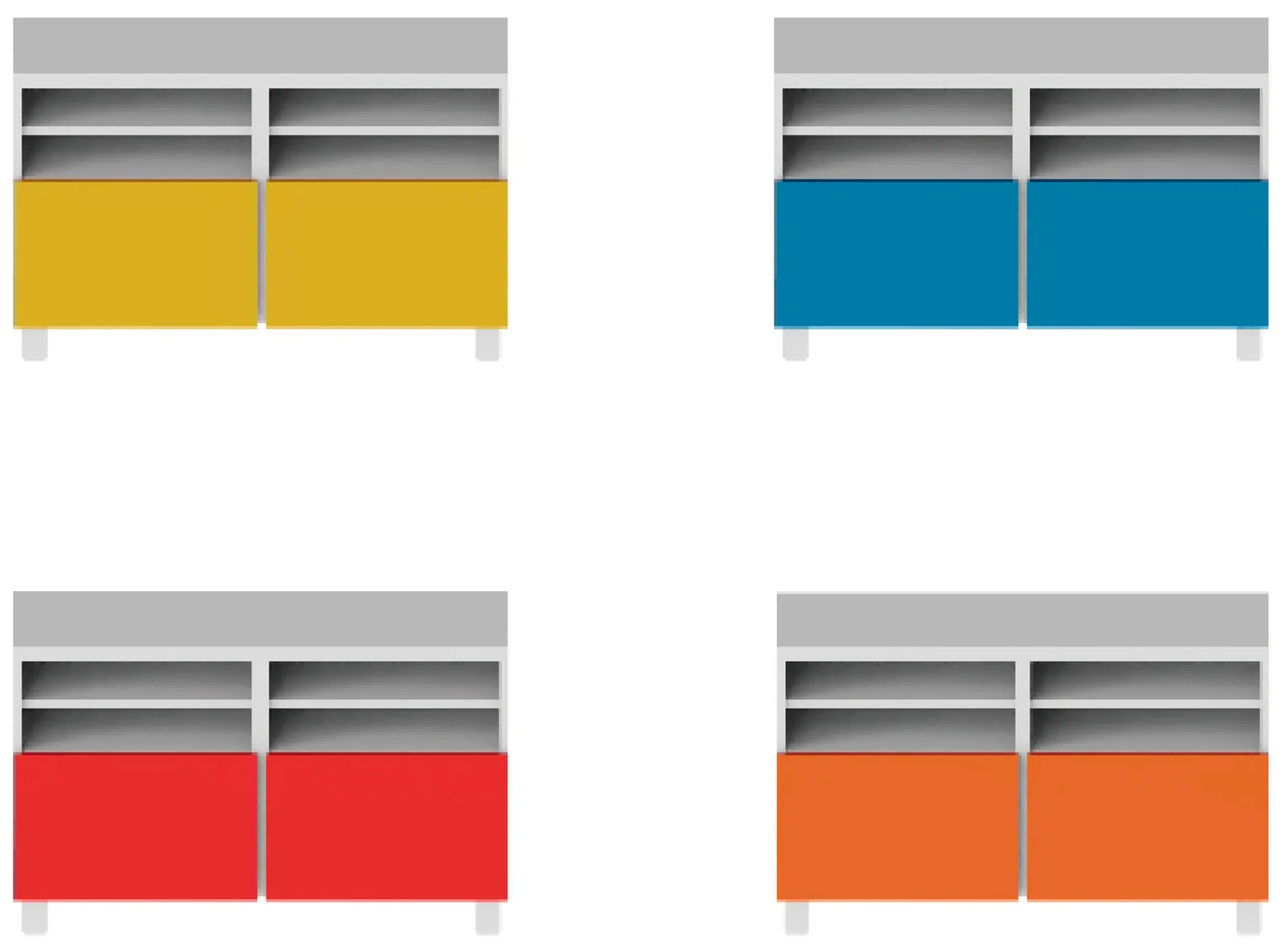
An example of one stimulus page.

**Figure 2:**
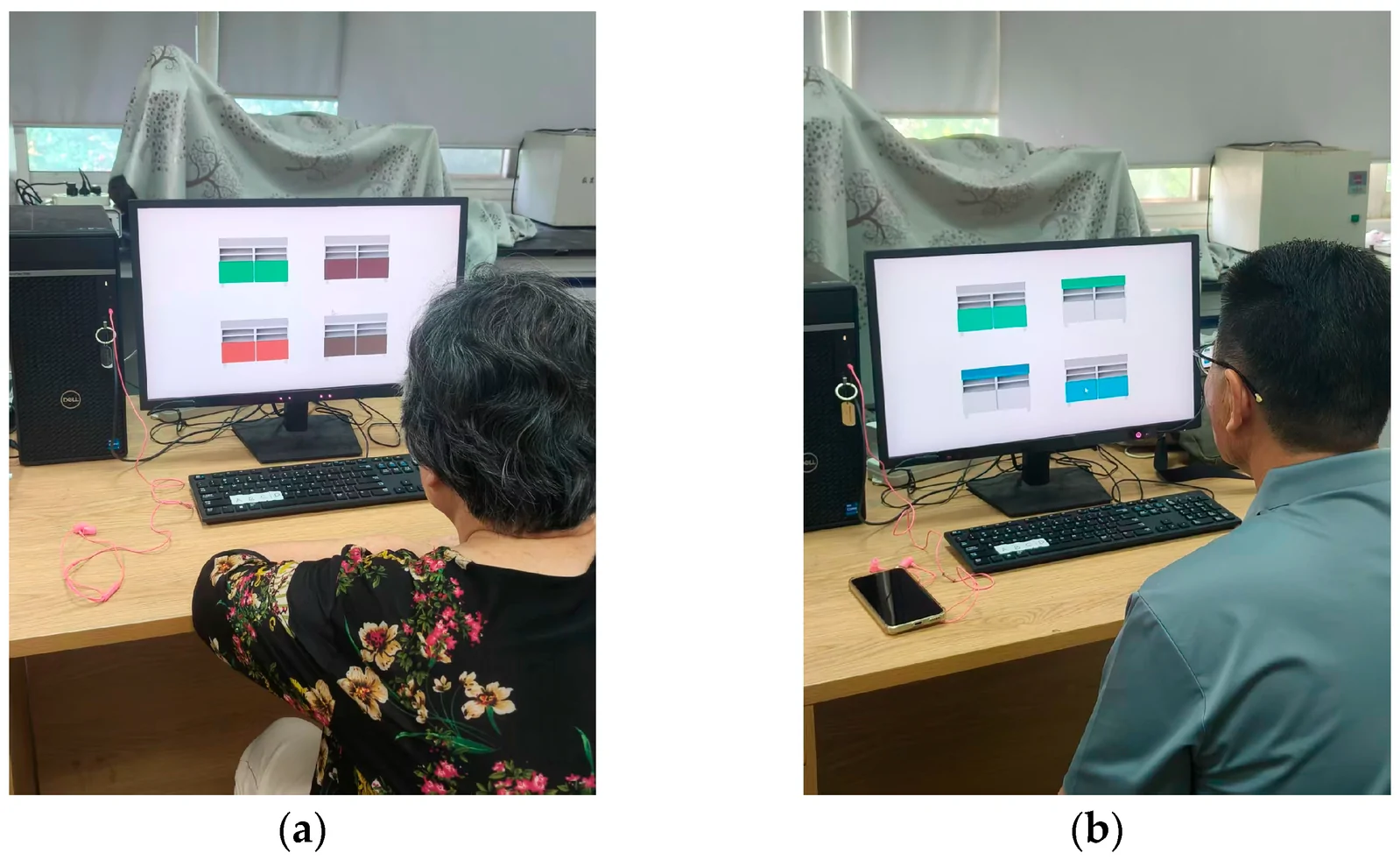
Eye-tracking experiment: (**a**) Experiment 1; (**b**) Experiment 2.

**Figure 3:**
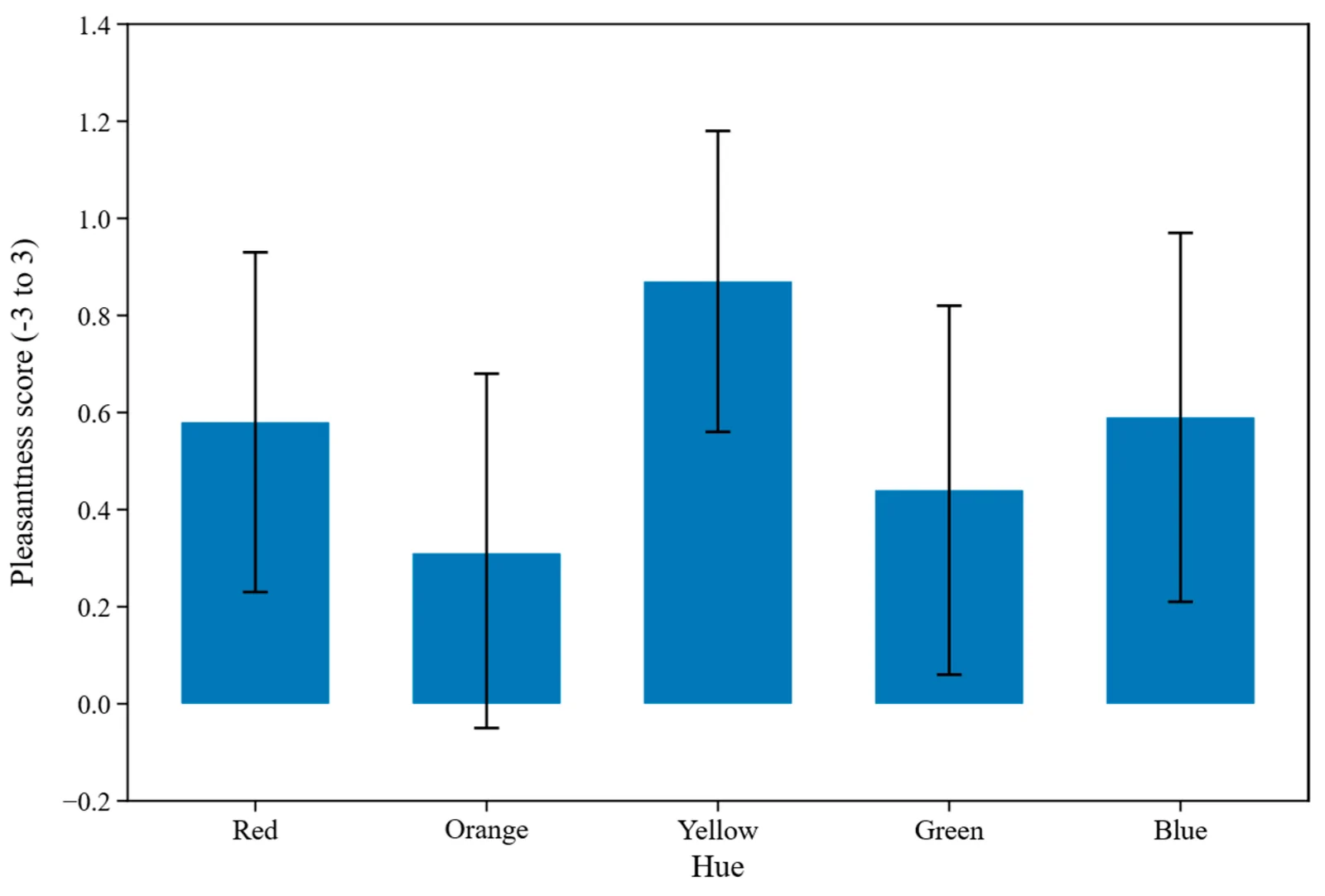
Marginal mean pleasantness ratings by hue in Experiment 1. Error bars represent 95% confidence intervals calculated from participant-level means (*n* = 35).

**Figure 4:**
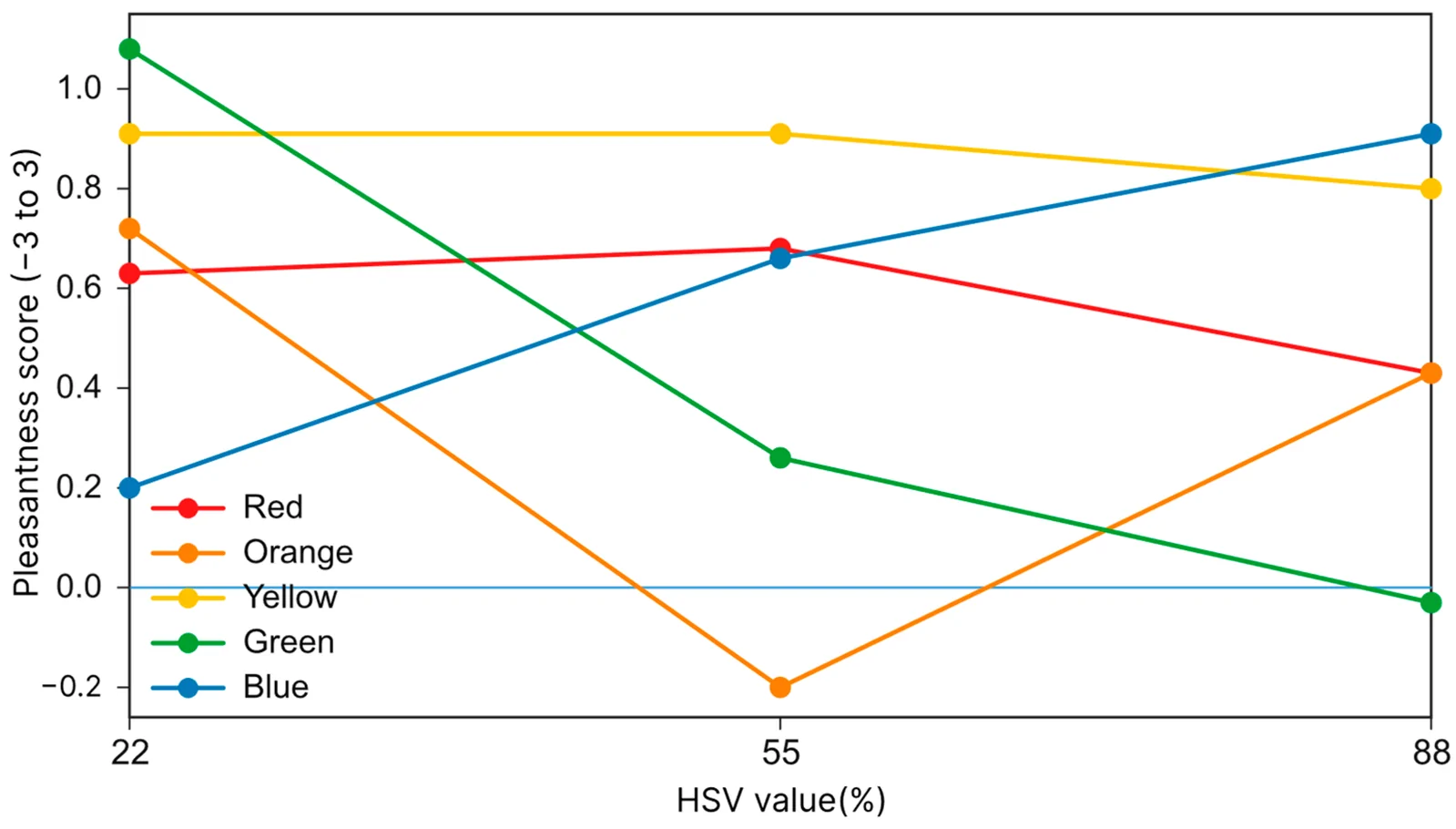
Subjective evaluation trends under hue × value conditions in Experiment 1. The light blue horizontal line represents the neutral baseline (pleasantness score = 0).

**Figure 5:**
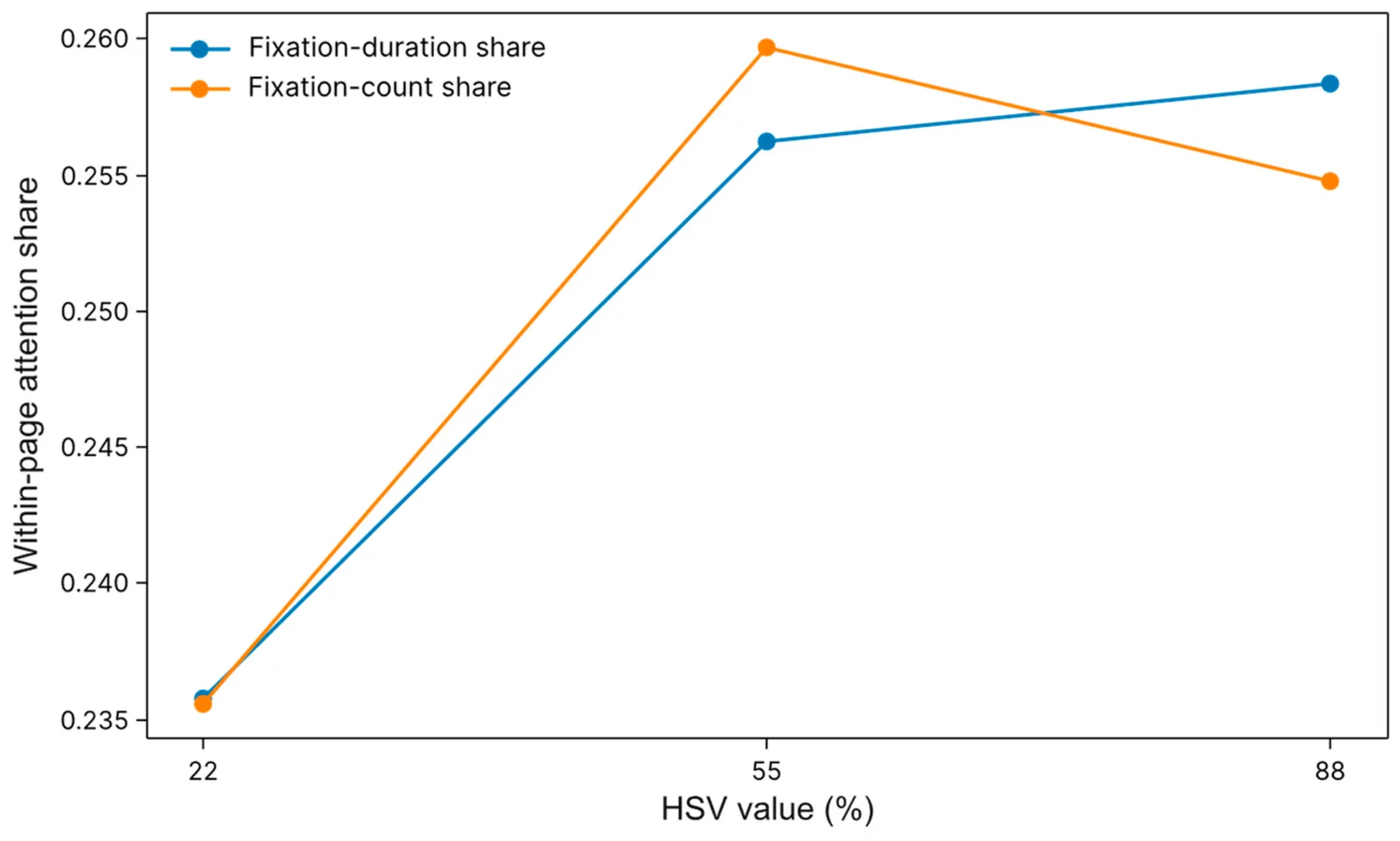
Marginal means of eye-movement indicators across value levels in Experiment 2.

**Figure 6:**
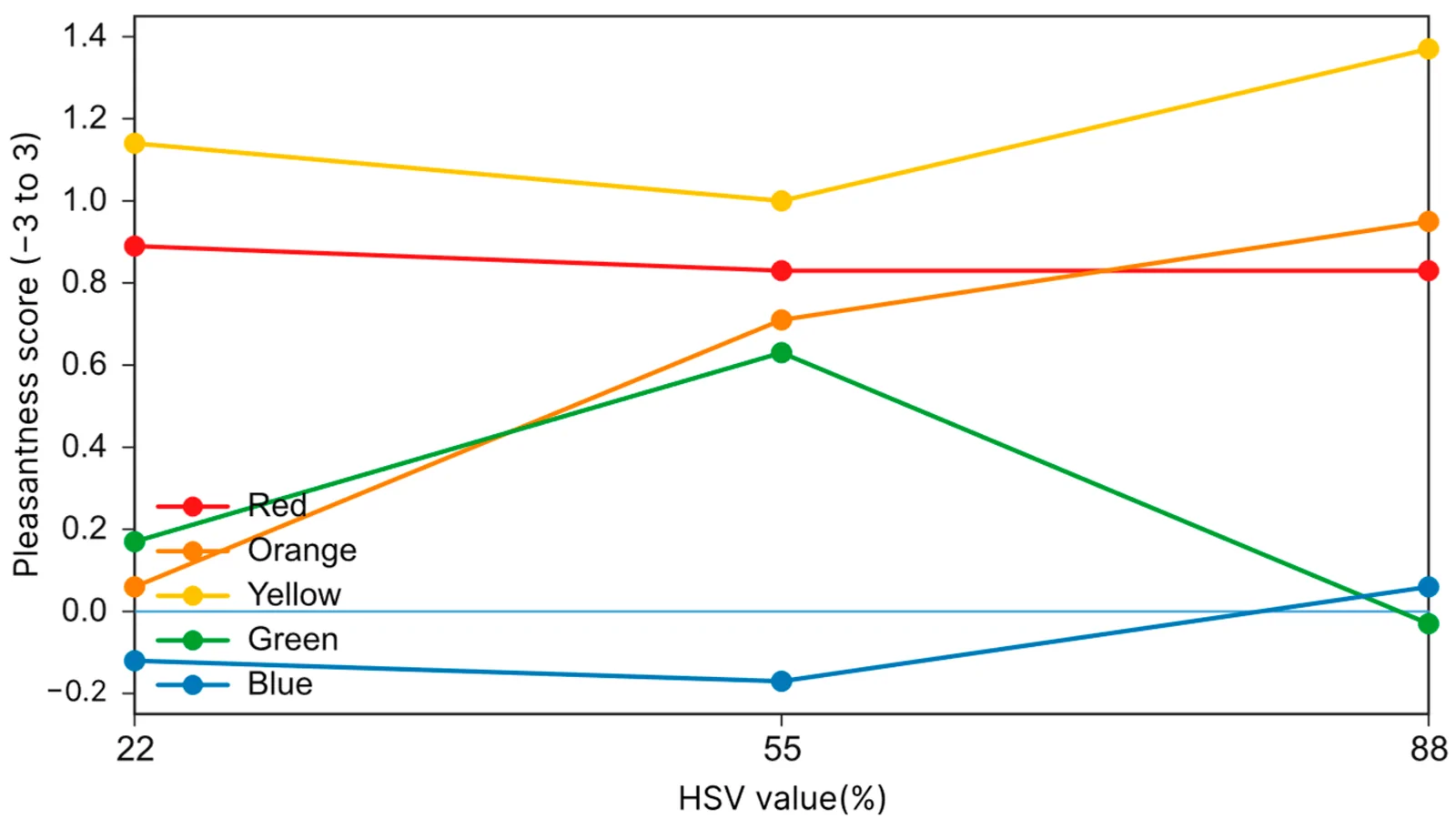
Subjective evaluation trends under auxiliary hue × value conditions in Experiment 2. The light blue horizontal line represents the neutral baseline (pleasantness score = 0).

**Figure 7:**
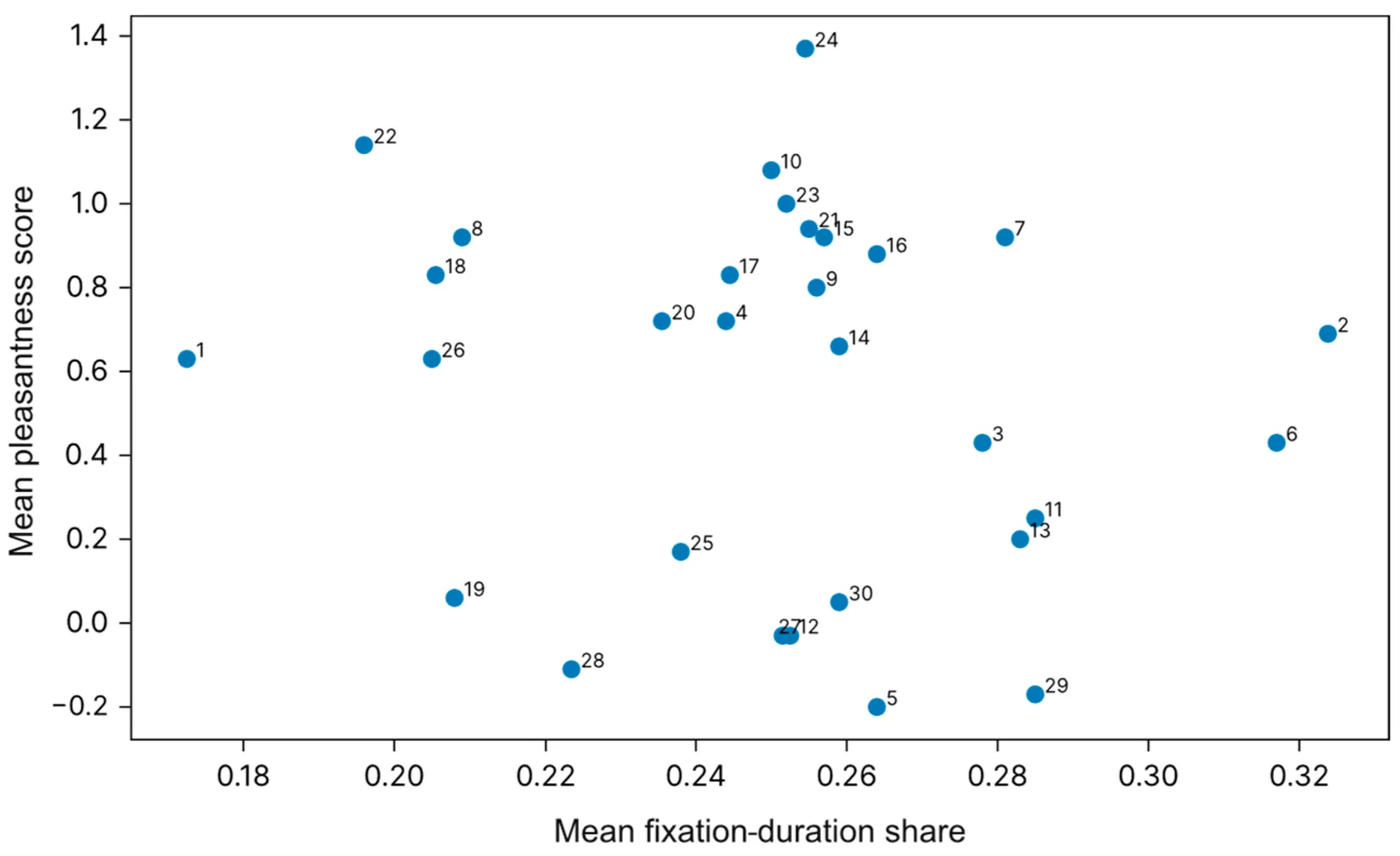
Relationship between subjective pleasure and eye-movement attention indicators.

**Figure 8:**
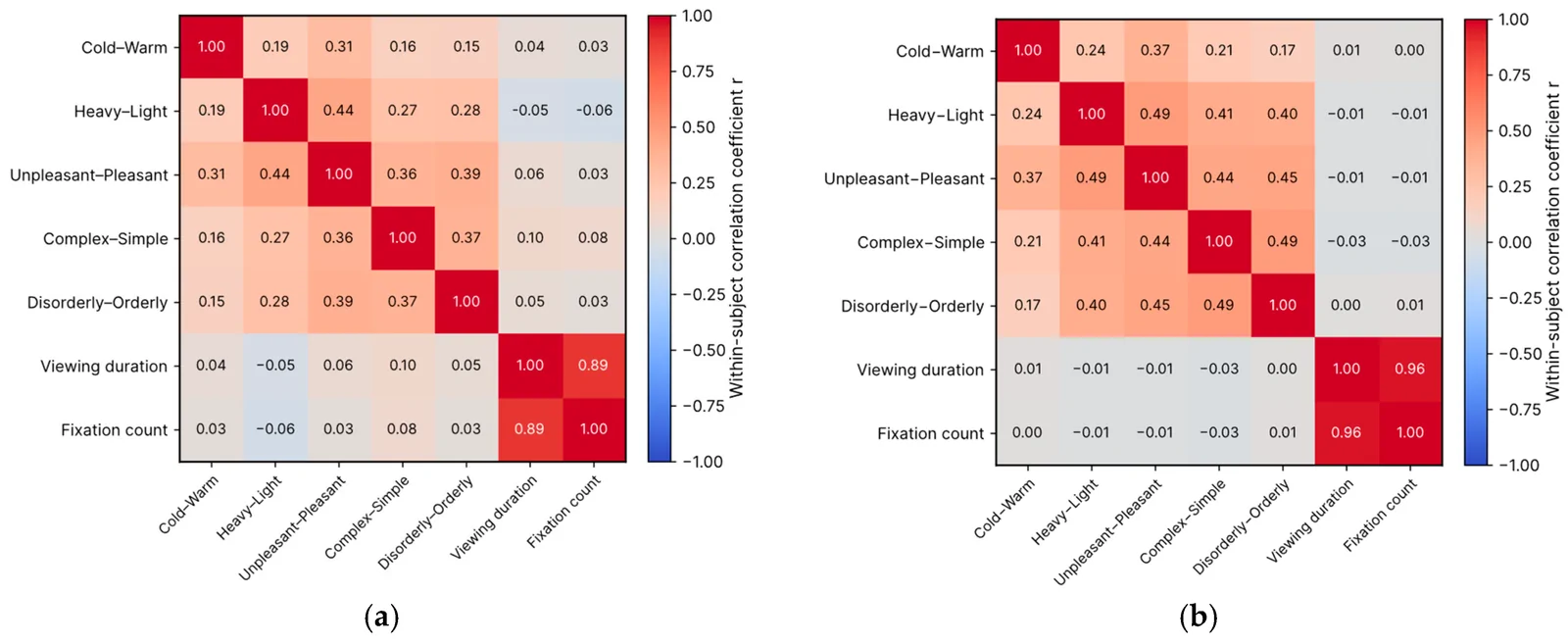
Within-subject correlation matrices between semantic evaluations and eye-movement indicators: (**a**) Experiment 1; (**b**) Experiment 2. All coefficients shown are within-subject correlation coefficients.

**Table 1:** Factors and stimulus design of the two experiments.

Experiment	Factors	Levels
Experiment 1	Hue	5 hues
	Value	3 value levels
Experiment 2	Dominant/secondary color relationship	2 two-color relationships
	Hue	5 hues
	Value	3 value levels

**Table 2:** Reliability and validity test results for the three-item aesthetic scale.

Experiment	Cronbach’s Alpha	McDonald’s Omega	KMO	Bartlett’s Test (*p*)	Single-Factor Explained Variance (%)	AVE
Experiment 1	0.884	0.929	0.616	<0.001	81.4%	0.814
Experiment 2	0.878	0.926	0.591	<0.001	80.9%	0.809

**Table 3:** Correlation analysis between subjective pleasure and eye-movement indicators.

Experiment	Eye-Movement Indicator	Within-Subject r	*p* Value	Condition-Mean Rho	*p* Value
Experiment 1	Viewing duration	0.057	0.193	0.095	0.736
Experiment 1	Fixation count	0.032	0.470	−0.162	0.565
Experiment 2	Viewing duration	−0.010	0.754	−0.036	0.851
Experiment 2	Fixation count	−0.010	0.749	−0.021	0.911

**Table 4:** Fixation-duration shares and regional effects.

Experiment	Top Panel	Open Shelf	Door Panel	Leg	Regional Effect
Experiment 1	34.5%	21.2%	20.4%	23.9%	F = 6.714, *p* = 0.001, ηp^2^ = 0.165
Experiment 2	29.8%	19.5%	32.6%	18.1%	F = 6.831, *p* = 0.001, ηp^2^ = 0.167

**Table 5:** Fixed effects of the linear mixed-effects model on subjective pleasantness.

Experiment	Fixed Effect	Wald χ^2^	df	*p*	Result
Experiment 1	Hue	12.43	4	0.014	Significant
	Value	4.01	2	0.135	Not significant
	Hue × Value	29.18	8	<0.001	Significant
Experiment 2	Hue	54.16	4	<0.001	Significant
	Value	0.23	2	0.891	Not significant
	Proportion	0.01	1	0.940	Not significant
	Hue × Value	20.35	8	0.009	Significant
	Hue × Proportion	24.39	4	<0.001	Significant
	Value × Proportion	6.54	2	0.038	Significant
	Hue × Value × Proportion	23.06	8	0.003	Significant

## Data Availability

The original contributions presented in this study are included in the article. Further inquiries can be directed to the corresponding author.
